# Physiological and molecular characterizations of the interactions in two cellulose-to-methane cocultures

**DOI:** 10.1186/s13068-017-0719-y

**Published:** 2017-02-07

**Authors:** Hongyuan Lu, Siu-Kin Ng, Yangyang Jia, Mingwei Cai, Patrick K. H. Lee

**Affiliations:** B5423-AC1, School of Energy and Environment, City University of Hong Kong, Tat Chee Avenue, Kowloon, Hong Kong

**Keywords:** Methanogenesis, Dark fermentation, Coculture, Cellulose degradation, Transcriptome, Metabolism

## Abstract

**Background:**

The interspecies interactions in a biomethanation community play a vital role in substrate degradation and methane (CH_4_) formation. However, the physiological and molecular mechanisms of interaction among the microbial members of this community remain poorly understood due to the lack of an experimentally tractable model system. In this study, we successfully established two coculture models combining the cellulose-degrading bacterium *Clostridium cellulovorans* 743B with *Methanosarcina barkeri* Fusaro or *Methanosarcina mazei* Gö1 for the direct conversion of cellulose to CH_4_.

**Results:**

Physiological characterizations of these models revealed that the methanogens in both cocultures were able to efficiently utilize the products produced by *C. cellulovorans* during cellulose degradation. In particular, the simultaneous utilization of hydrogen, formate, and acetate for methanogenesis was observed in the *C. cellulovorans*–*M. barkeri* cocultures, whereas monocultures of *M. barkeri* were unable to grow with formate alone. Enhanced cellulose degradation was observed in both cocultures, and the CH_4_ yield of the *C. cellulovorans*–*M. barkeri* cocultures (0.87 ± 0.02 mol CH_4_/mol glucose equivalent) was among the highest compared to other coculture studies. A metabolic shift in the fermentation pattern of *C. cellulovorans* was observed in both cocultures. The expression levels of genes in key pathways that are important to the regulation and metabolism of the interactions in cocultures were examined by reverse transcription-quantitative PCR, and the expression profiles largely matched the physiological observations.

**Conclusions:**

The physiological and molecular characteristics of the interactions of two CH_4_-producing cocultures are reported. Coculturing *C. cellulovorans* with *M. barkeri* or *M. mazei* not only enabled direct conversion of cellulose to CH_4_, but also stabilized pH for *C. cellulovorans*, resulting in a metabolic shift and enhanced cellulose degradation. This study deepens our understanding of interspecies interactions for CH_4_ production from cellulose, providing useful insights for assembling consortia as inocula for industrial biomethanation processes.

**Electronic supplementary material:**

The online version of this article (doi:10.1186/s13068-017-0719-y) contains supplementary material, which is available to authorized users.

## Background

Biomethanation, a natural biological process by which organic materials are transformed into biogas, can be deployed for waste treatment and sustainable energy production [1, 2]. This process has been widely applied in municipal sewage treatment to not only effectively reduce the volume and odor of volatile solids, but also produce methane (CH_4_) as an energy resource to power treatment facilities [3]. As the worldwide demand for renewable energy increases, biomethanation of organic materials has an important role to play in our energy future [4].

The biomethanation process in nature relies on the microbial interactions between three main metabolic groups of anaerobes: fermentative, acetogenic, and methanogenic microorganisms [5–7]. The first two groups decompose complex organic matters to acetate, hydrogen (H_2_), and carbon dioxide (CO_2_), which are the key precursors for methanogenesis. Methanogens further convert these metabolites to CH_4_ by two major routes: the acetoclastic and CO_2_ reduction pathways [8]. Although methanogens are obligately dependent on the first two metabolic groups to supply substrates for growth, the two methanogenesis pathways can in turn affect their activities. First, H_2_ production by some bacteria is thermodynamically unfavorable; therefore, their growth is contingent on the CO_2_-reducing methanogens to maintain a low H_2_ partial pressure [9]. For example, the maintenance of a very low concentration of H_2_ in the ecosystem by the methanogens is essential for the catabolism of fatty acids by the obligate proton-reducing acetogenic bacteria *Desulfovibrio vulgaris* [10, 11]. Second, the consumption of acetate by acetate-utilizing methanogens can help to maintain a pH close to neutral to support optimal metabolic activities of other members [2]. These illustrated interdependent relationships form the basis of many interactions that occur in a biomethanation community.

Because cellulosic materials are commonly found in nature, methanogens often exist concomitantly with cellulose-fermenting bacteria in anaerobic habitats, such as sediments, sewage digesters, and landfills [12–14]. Cellulose-fermenting bacteria hydrolyze the insoluble cellulose into end products—such as organic acids, CO_2_, and H_2_—which become carbon and energy sources for other members, including methanogens, within the microbial community. Because of this substrate dependency, the interactions between the cellulolytic bacteria and methanogens play a crucial role in shaping a biomethanation community. In order to gain insights into the metabolic functions of the cellulolytic-methanogenic communities, efforts have been made to study the interactions between cellulose-fermenting bacteria and H_2_/formate/acetate-consuming methanogens in artificially constructed cultures. For example, Laube and Martin [15] studied cocultures of *Acetivibrio cellulolyticus*–*Methanosarcina barkeri* and *M. barkeri*-*Desulfovibrio* sp., as well as a triculture integrating *A. cellulolyticus*, *M. barkeri*, and *Desulfovibrio* sp. Their results showed that the methanogen was able to utilize the H_2_ and acetate produced by the cellulose-fermenting bacteria for CH_4_ production, resulting in improved CH_4_ production and a faster fermentation rate in the triculture. Nakashimada et al. [16] investigated cocultures of the anaerobic fungi *Neocallimastix frontalis* with a formate- and H_2_-utilizing methanogen (*Methanobacterium formicicum*) or an acetoclastic methanogen (*Methanosaeta concilii*), as well as a triculture incorporating *N. frontalis*, *M. formicicum*, and *M. concilii.* Their results demonstrated that whereas the coculture of *N. frontalis*–*M. formicicum* utilized formate and H_2_ and the coculture of *N. frontalis*-*M. concilii* utilized acetate for CH_4_ production, the triculture of *N. frontalis*, *M. formicicum*, and *M. concilii* was able to use formate, H_2_, and acetate for CH_4_ production. Robert et al. [17] investigated interspecies H_2_ transfer by employing cocultures of fibrolytic bacteria and the H_2_-utilizing colonic methanogen *Methanobrevibacter smithii* and observed that H_2_ utilization by the methanogen induced a metabolic shift in the cellulolytic strain. Sasaki et al. [18] incorporated *C. clariflavum* CL-1 and the hydrogenotrophic methanogen *Methanothermobacter thermautotrophicus* ∆H under thermophilic conditions. They reported that the cellulose degradation efficiency and cell density of *C. clariflavum* CL-1 were significantly higher in the coculture than in the monoculture. Bauchop et al. [19] employed a rumen anaerobic fungus with a consortium of rumen methanogens for methanogenesis from cellulose and observed a metabolic shift in the fungus.

Although the aforementioned coculture and triculture studies have all demonstrated that CH_4_ can be successfully produced from products of cellulose hydrolysis by various methanogens, the simultaneous utilization of H_2_, formate, and acetate by a single methanogen in a coculture has not yet been reported. In addition, the molecular mechanism of the interactions between fermentative cellulose degraders and methanogens is also unclear; however, such an understanding is essential in order to shed light on key cellular regulation and metabolism during coculturing. For instance, gene expression of key pathways related to the metabolic shift in cellulolytic bacteria and the activity of methanogen is of great importance to understand the carbon and electron flows between these two organisms. In this study, we examined whether the metabolic versatile methanogen *Methanosarcina barkeri* Fusaro—which is potentially capable of utilizing H_2_, formate, and acetate for methanogenesis—can form a coculture with the cellulose-degrading bacterium *Clostridium cellulovorans* 743B, which is capable of producing all three methanogenesis precursors (H_2_, formate, and acetate) as major fermentation metabolites. Meanwhile, *Methanosarcina mazei* Gö1, which possesses the ability to utilize H_2_ and acetate but not formate for methanogenesis, was also employed for coculturing with *C. cellulovorans* to enable comparison with the *C. cellulovorans*–*M. barkeri* cocultures. The genomes of *C. cellulovorans*, *M. barkeri*, and *M. mazei* [20–22] have all been fully sequenced, making the two cocultures genetically tractable in order to understand the molecular mechanisms of the interactions. The physiology of the two coculture models was characterized, and the expression levels of genes in key pathways in cocultures and monocultures were analyzed and compared. Overall, the results of this study provide insights into the interactions between the cellulolytic bacterium and the methanogens, and a comprehensive understanding of these interactions is crucial for engineering synthetic consortia for large-scale biomethanation processes to produce energy from renewable cellulosic biomass.

## Methods

### Cultures and growth conditions

The dark fermentative bacterium *C. cellulovorans* (ATCC# 35296) was purchased from the American Type Culture Collection (ATCC, VA, USA), whereas the two methanogens—*M. barkeri* (DSM# 804) and *M. mazei* (DSM# 3647)—were purchased from the German Collections of Microorganisms and Cell Cultures (DSMZ, Germany). *C. cellulovorans*, *M. barkeri*, and *M. mazei* were first grown in the respective media recommended by the culture collections to revive the lyophilized cells. Subsequently, active inoculum (5% vol/vol) of *C. cellulovorans* was transferred to a defined medium (DCB-1) with 3 g/L of cellulose as previously described [23] with the following modifications: 2 g/L of yeast extract, 10 g/L of sodium chloride, and 9.5 g/L of magnesium sulfate were added, and CO_2_/N_2_ (20%:80%) filled the headspace. Active inocula (10% vol/vol) of *M. barkeri* and *M. mazei* were transferred to a defined high-salt medium [24] with 50 mM of acetate and CO_2_/N_2_ (20%:80%) in the headspace. After inoculation, the methanogens grown with acetate required about 6 months of acclimation before noticeable growth occurred, but thereafter the cultures could be routinely propagated every 2 weeks.

All monoculture and coculture experiments were carried out in 160-mL serum bottles with 100 mL of the DCB-1 medium as described above and incubated at 35 °C without shaking. Monocultures of *C. cellulovorans* were grown with 3 g/L of cellulose, whereas monocultures of *M. barkeri* and *M. mazei* were grown with 50 mM of acetate. For coculture experiments, 3 g/L of cellulose was amended as the only substrate, and *C. cellulovorans* and *M. barkeri* were inoculated at a cell ratio of 1.4:1, whereas *C. cellulovorans* and *M. mazei* were inoculated at a cell ratio of 1.7:1. The inoculum for each experiment was obtained from the mid-exponential growth phase of the respective monocultures. To examine whether monocultures of *M. barkeri* can grow with formate alone, active inoculum of *M. barkeri* grown with acetate was transferred to the DCB-1 medium and high-salt medium amended with 50 mM of formate. In addition, to evaluate whether the presence of other methanogenesis precursors affected the consumption of formate by *M. barkeri*, active inoculum of *M. barkeri* grown with acetate was transferred to the DCB-1 medium amended with all three methanogenesis precursors (10 mM of formate, 3 mM of acetate, and 0.95 mmol of H_2_), and to the DCB-1 medium with 3 mM of acetate and 0.95 mmol of H_2_ as control. These conditions mimicked the concentrations of the three methanogenesis precursors in the *C. cellulovorans* monocultures at mid-exponential growth phase. All experiments were performed in triplicate.

### Analytical analyses

The total volume of gas accumulated at each time point was measured. A needle connected to a disposable syringe was inserted through the stopper into the headspace when taking measurement. The volume in the calibrated syringe after plunger displacement was the gas accumulated. The concentrations of H_2_ and CH_4_ were sampled using a gas-tight syringe (Hamilton, NV, USA) and analyzed using a gas chromatograph (GC-2010, Shimadzu, Japan) equipped with a thermal conductivity detector and a flame ionization detector. The column (30 m × 0.53 mm inner diameter) for H_2_ detection was a 5A molecular sieve (Restek, PA, USA) and the column (30 m × 0.53 mm inner diameter) for CH_4_ detection was a Rt–QS-BOND column (Restek, PA, USA), both with helium as a carrier gas. The column temperature was 35 °C, the detector temperature was 200 °C, and the injector temperature was 120 °C for both analyses. The CO_2_ produced by *C. cellulovorans* [25] was not determined since excess CO_2_ was provided by filling the headspace of the culture bottles with CO_2_/N_2_ (20%:80%) and 2.5 g/L of sodium bicarbonate was added as part of the DCB-1 medium. The concentrations of gas (*C*_*G*_) were converted to total mole (*n*) of H_2_ or CH_4_ in each serum bottle using the corresponding Henry’s constant (*H*) [26] according to the following material balance: $n = C_{\text{G}} V_{\text{G}} + C_{\text{G}} H V_{\text{L}}$, where *V*_G_ and *V*_L_ are the gas and liquid volumes in a serum bottle, respectively. Two mLs of sample were withdrawn from each culture bottle to measure pH, the concentrations of metabolic products and cellulose, and the cell density as described previously [27]. Glucose equivalent is a measure of the amount of reducing sugars present in a sugar product, relative to glucose [28].

### Cell morphology analysis

The morphology of cells in the cocultures and monocultures was visualized by scanning electron microscopy (SEM). SEM analysis was performed as previously described [29] with some modifications. Samples were collected from biological triplicate during the mid-exponential growth phase according to the amount of H_2_ or CH_4_ produced and pooled prior to cell fixation. Cells were fixed for 24 h at 4 °C in 2% vol/vol glutaraldehyde in 0.1 M sodium cacodylate buffer (pH 7.2). Subsequently, the cells were washed with 0.1, 0.05, and 0.025 M of cacodylate buffers for 15 min each. Samples were then dehydrated through a gradient of ethanol concentrations (50, 70, and 90% vol/vol) for 15 min each, then washed three times with 100% ethanol and acetone for 15 min each. Ethanol–acetone dehydrated samples were critical-point dried in liquid CO_2_ with a Bal-Tec CPD 030 critical-point drier (Bal-Tec, Balzers, Liechtenstein). With the use of carbon tape, the samples were stuck on aluminum stubs. The dehydrated samples were observed under a Philips XL30 ESEM FEG environmental SEM (Philips Electronics, Netherlands) after being sputter coated with gold palladium using a Bal-Tec SCD 050 sputter coater (Bal-Tec, Balzers, Liechtenstein).

### Cell density analysis

Genomic DNA extraction and absolute quantification of cell number of the respective organisms in each culture with quantitative PCR (qPCR) were performed as described previously [27]. *C. cellulovorans* was quantified by targeting its cellulase gene (Gene ID: 9607758) with forward primer 5′-ACAGCGCAAGATGGCTTCTA-3′ and reverse primer 5′-GCTGTAGCTCCCCATTGAGT-3′, *M. barkeri* by its formate dehydrogenase subunit alpha gene (Gene ID: 3625978) with forward primer 5′-TCGGACCCGGATCTAAACAA-3′ and reverse primer 5′-ATTGGTCTGGGTCCCGTTCT-3′, and *M. mazei* by its methyl-coenzyme M reductase gene (Gene ID: 1479582) with forward primer 5′-ATGCAGCAGATGTGGGATGAC-3′ and reverse primer 5′-CGACCATCATTTCCTGAACCA-3′. One copy of the target gene was found in each respective genome. All primers in this study were designed using Primer Express 3.0 (Applied Biosystems, CA, USA) and their specificity was verified.

### RNA extraction

Duplicate monocultures or cocultures were prepared for mRNA relative quantification analysis. Cells were collected during the mid-exponential growth phase, and total RNA was extracted using the protocol described previously [30]. The purity and concentration of the total RNA were determined by a Nano Drop 2000 spectrophotometer (Thermo Fisher Scientific, MA, USA). For all samples, A_260_/A_280_ ratios ranged from 2.0 to 2.1, and concentrations were above 91 ng/µL. The integrity and quality of the total RNA were further assessed on a bioanalyzer 2100 (Agilent, CA, USA) with the Agilent RNA 6000 Pico kit (Agilent, CA, USA) according to the manufacturer’s instructions. Only samples with an RNA integrity number (RIN) above 7.5 were used for downstream analyses.

### Reverse transcription (RT)-qPCR

To examine the cellular regulation of key pathways of the cocultures and monocultures, 37 genes were selected and analyzed by RT-qPCR using specific primers (Additional file 1: Table S1) for relative mRNA quantification. The genes of *C. cellulovorans* are designated as “Clocel,” *M. barkeri* as “Mbar A,” and *M. mazei* as “MM.” Total RNA was reverse-transcribed into complementary DNA (cDNA) with random hexamers using the SuperScript III (Invitrogen, CA, USA) reverse transcriptase according to the manufacturer’s instructions. For negative reverse transcriptase controls to verify the absence of genomic DNA, diethylpyrocarbonate-treated water replaced the reverse transcriptase. Amplification of the synthesized cDNA (two technical replicates per biological replicate) and negative controls was performed on a StepOne Plus Real-Time PCR System (Applied Biosystems, CA, USA) using the PowerUp™ SYBR Green Master Mix (Applied Biosystems, CA, USA) according to the manufacturer’s instructions and default thermal-cycling conditions.

Comparative threshold (Ct) differences between cocultures and monocultures were calculated using averages of quadruplicate samples. The fold difference for each target gene was calculated using the 2^−∆∆Ct^ method [31] (reported as ratio of coculture/monoculture). Because peptidyl-prolyl isomerase (cyclophilin) is considered a stable housekeeping gene [32, 33], and our previous experiments with *C. cellulovorans* [30] validated the expression of this gene was unaffected by experimental treatment, peptidyl-prolyl isomerase was used as an internal control gene for normalization for *C. cellulovorans*. On the other hand, the glyceraldehyde dehydrogenase (*gap*) gene of *M. barkeri* and *M. mazei* [34] was used as an internal control gene for normalization. Specific primers for the three internal control genes are listed in Additional file 1: Table S2. The statistical significance of the expression ratio of each gene between two conditions (cocultures versus monocultures) was analyzed using the Student’s *t* test. The standard deviation of the fold changes across replicates of each gene was on average equal to an absolute value of 0.6 fold. An absolute value of the fold change ≥1.2 (twice the average standard deviation across replicates) and a *p* value <0.05 were set as thresholds to identify genes that were differentially expressed between cocultures and monocultures. Upregulation (a positive expression ratio) refers to a higher relative molar concentration of the transcripts of a particular gene of *C. cellulovorans*, *M. barkeri*, or *M. mazei* in cocultures relative to the respective monocultures, and downregulation (a negative expression ratio) refers to a lower relative molar concentration of transcripts in cocultures. The expression ratios of the differentially expressed genes (|fold change| ≥1.2 and *p* value <0.05) are shown as heat maps.

## Results

### Methanogenesis and cellulose utilization

**Fig. 1:**
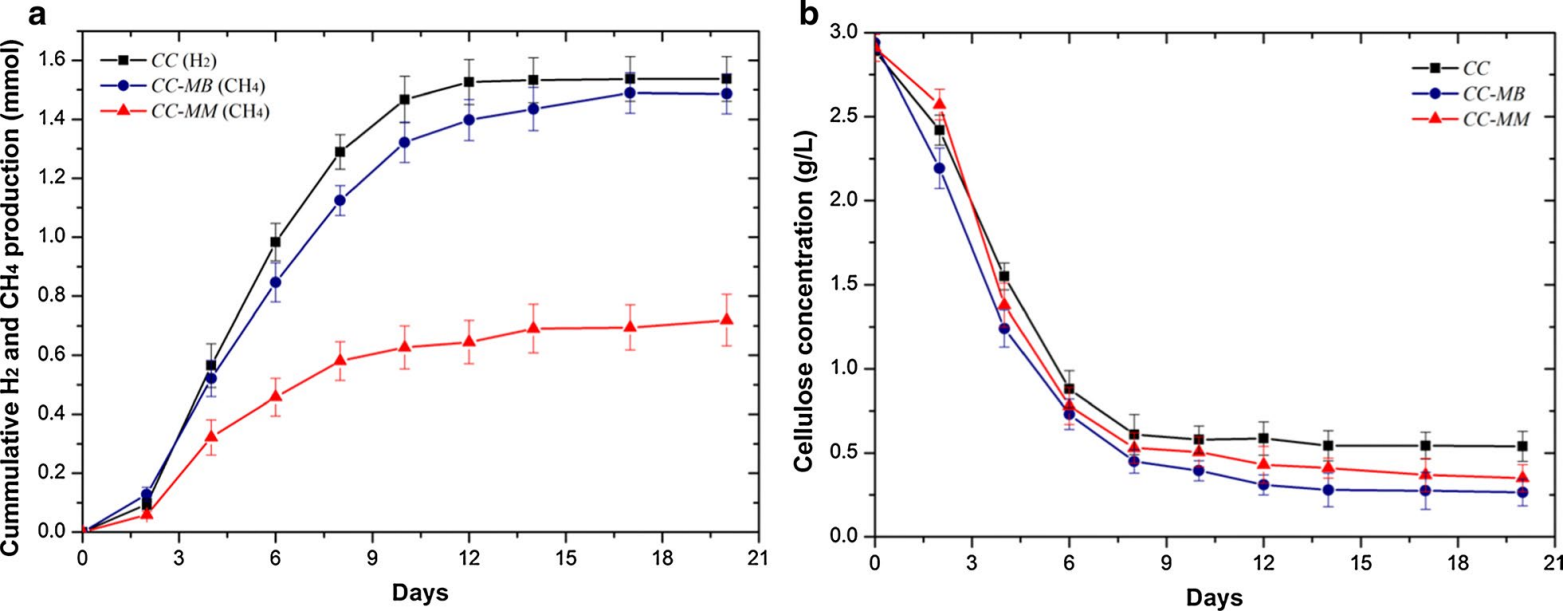
**a** Comparison of H_2_ and CH_4_ production and **b** cellulose degradation for cocultures of *C. cellulovorans*–*M. barkeri* (*CC*-*MB*) and *C. cellulovorans*–*M. mazei* (*CC*-*MM*) and monocultures of *C. cellulovorans* (*CC*). Each data point is an average of biological triplicate and *error bars* represent one standard deviation

CH_4_ and H_2_ production by cocultures of *C. cellulovorans*–*M. barkeri* and *C. cellulovorans*–*M. mazei* and by monocultures of *C. cellulovorans* were examined by amending 3 g/L of cellulose as the sole substrate. Coculturing *C. cellulovorans* with either *M. barkeri* or *M. mazei* both led to methanogenesis during the cultivation period, and linear CH_4_ production was observed from day 2 onwards (Fig. 1a). The total duration of CH_4_ production for *C. cellulovorans*–*M. barkeri* was longer than that for *C. cellulovorans*–*M. mazei* (17 versus 10 days), and the final total amount of CH_4_ produced in *C. cellulovorans*–*M. barkeri* was substantially higher than that produced in *C. cellulovorans*–*M. mazei* (1.5 ± 0.07 mmol versus 0.7 ± 0.09 mmol). Monocultures of *M. barkeri* and *M. mazei* grown on 50 mM acetate produced 2.8 ± 0.1 mmol and 2.5 ± 0.09 mmol of CH_4_, respectively (Additional file 1: Figure S1). On the other hand, 0.3 ± 0.01 mmol of CH_4_ accumulated in monocultures of *M. barkeri* grown with 10 mM formate, 3 mM acetate, and 0.95 mmol H_2_, whereas 0.2 ± 0.01 mmol of CH_4_ accumulated in monocultures of *M. barkeri* grown with 3 mM acetate and 0.95 mmol H_2_. In these monocultures, H_2_ was depleted gradually during the cultivation period (Additional file 1: Figures S2, S3). No CH_4_ was observed in the monocultures of *M. barkeri* grown with formate alone. As opposed to CH_4_ production, 1.5 ± 0.08 mmol of H_2_ accumulated in monocultures of *C. cellulovorans*, whereas no H_2_ was detected in the cocultures throughout the cultivation period.

Cellulose degradation was observed in both cocultures and monocultures of *C. cellulovorans*. Coculturing *C. cellulovorans* with either *M. barkeri* (+13.8%) or *M. mazei* (+8.9%) both resulted in enhanced cellulose degradation relative to monocultures of *C. cellulovorans* (Fig. 1b). At the end of the incubation period, a small amount of cellulose remained. This is likely due to the acidic pH, as a result of volatile fatty acids (VFAs) accumulation, inhibiting the metabolic activity of the dark fermenters [35]. No growth or cellulose degradation was observed in the controls of *M. barkeri* and *M. mazei* monocultures grown on cellulose. Corresponding to the higher CH_4_ production and more complete cellulose degradation, the CH_4_ yield and production rate of *C. cellulovorans*–*M. barkeri* were nearly two times higher than those of *C. cellulovorans*–*M. mazei* after taking into account the amount of cellulose consumed (Table 1). Together, these results indicate that coculturing *C. cellulovorans* with either *M. barkeri* or *M. mazei* not only enabled CH_4_ production from cellulose but also enhanced cellulose degradation in comparison to monocultures of *C. cellulovorans*. *C. cellulovorans*–*M. barkeri* exhibited a stronger ability of methanogenesis than *C. cellulovorans*–*M. mazei* in terms of CH_4_ yield and transformation rate.

**Table 1:** Mass and rate of cellulose consumption, and yield and rate of H_2_ and CH_4_ production

Cultures	Cellulose consumed (mg)	H_2_ yield (mol H_2_/mol glucose equivalent)	CH_4_ yield (mol CH_4_/mol glucose equivalent)	Cellulose degradation rate (mg/day)	H_2_ production rate (mmol/day)	CH_4_ production rate (mmol/day)
*C. cellulovorans*	235 ± 8	1.02 ± 0.03	–	117 ± 4	0.077 ± 0.004	–
*C. cellulovorans*–*M. barkeri*	267 ± 16	–	0.87 ± 0.02	133 ± 8	–	0.074 ± 0.003
*C. cellulovorans*–*M. mazei*	256 ± 7	–	0.44 ± 0.04	128 ± 4	–	0.036 ± 0.004

Data are the average (±one standard derivation) of biological triplicate

### Fermentation products and pH

**Fig. 2:**
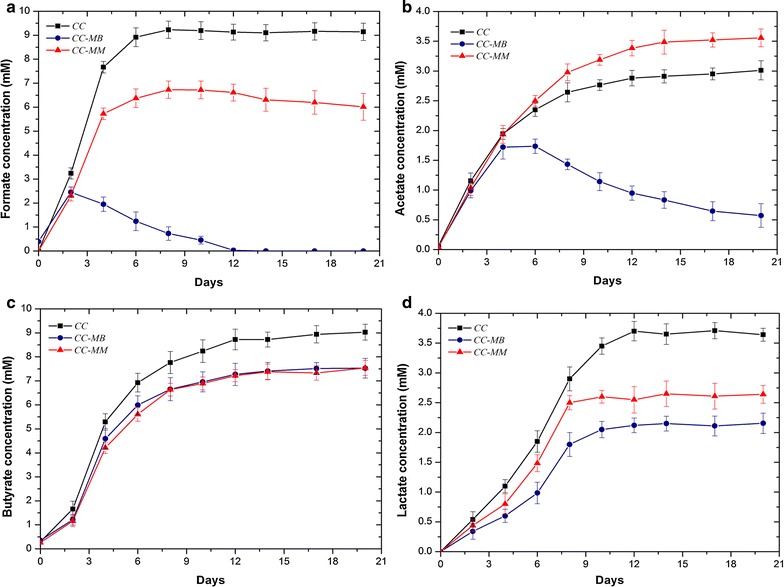
Comparison of VFA concentrations (**a** formate, **b** acetate, **c** butyrate, **d** lactate) for cocultures of *C. cellulovorans*–*M. barkeri* (*CC*-*MB*) and *C. cellulovorans*–*M. mazei* (*CC*-*MM*) and monocultures of *C. cellulovorans* (*CC*). Each data point is an average of biological triplicate and *error bars* represent one standard deviation

Monocultures of *C. cellulovorans* mainly fermented cellulose to H_2_, CO_2_, and VFAs, including formate, acetate, butyrate, and lactate (Additional file 1: Table S3). Production of VFAs by *C. cellulovorans* monocultures increased gradually to maximum accumulated concentrations of 9.1 ± 0.4 mM of formate, 3.0 ± 0.2 mM of acetate, 9.0 ± 0.3 mM of butyrate, and 3.6 ± 0.1 mM of lactate (Fig. 2). However, the presence of methanogens in cocultures led to VFAs consumption and induced a change in the fermentation pattern of *C. cellulovorans*, resulting in different VFA concentrations in cocultures relative to the *C. cellulovorans* monocultures. For the cocultures of *C. cellulovorans*–*M. barkeri*, formate and acetate only transiently accumulated during the cultivation period. Formate concentration reached its peak of 2.5 ± 0.2 mM on day 2, then decreased rapidly until depletion on day 12 (Fig. 2a). Similarly, the acetate concentration also decreased progressively until near depletion after reaching its maximum concentration of 1.7 ± 0.1 mM on day 6 (Fig. 2b). The depletion of formate and acetate indicates their utilization via methanogenesis by *M. barkeri*. In contrast, net reduction in the concentrations of butyrate and lactate concentrations was not observed during the cultivation period. However, the final concentrations of butyrate and lactate were both lower in *C. cellulovorans*–*M. barkeri* than in the *C. cellulovorans* monocultures (Fig. 2c, d). On the other hand, only 1.5 ± 0.4 mM of formate and insignificant amount of acetate were consumed in monocultures of *M. barkeri* grown with 10 mM formate, 3 mM acetate, and 0.95 mmol H_2_. Similarly, insignificant amount of acetate was consumed in monocultures of *M. barkeri* grown with 3 mM acetate and 0.95 mmol H_2_ (Additional file 1: Figures S2, S3). For the cocultures of *C. cellulovorans*–*M. mazei*, neither formate nor acetate depletion was observed during the cultivation period. However, whereas the final formate concentration in *C. cellulovorans*–*M. mazei* was lower than in *C. cellulovorans* monocultures, the final acetate concentration in *C. cellulovorans*–*M. mazei* was higher by 18% (Fig. 2a, b), and the final concentrations of butyrate and lactate were also lower in *C. cellulovorans*–*M. mazei* (Fig. 2c, d).

**Fig. 3:**
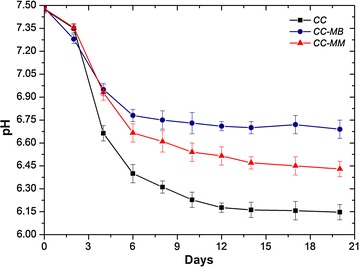
Comparison of pH for cocultures of *C. cellulovorans*–*M. barkeri* (*CC*-*MB*) and *C. cellulovorans*–*M. mazei* (*CC*-*MM*) and monocultures of *C. cellulovorans* (*CC*). Each data point is an average of biological triplicate and *error bars* represent one standard deviation

The pH of the *C. cellulovorans* monocultures decreased rapidly from the initial pH of 7.5 ± 0.02 to 6.1 ± 0.05, but the change in the cocultures of *C. cellulovorans*–*M. barkeri* and *C. cellulovorans*–*M. mazei* was smaller and more gradual (Fig. 3). The more stabilized pH of the cocultures compared to the *C. cellulovorans* monocultures is likely due to the consumption of VFAs by the methanogens and the change in fermentation pattern of *C. cellulovorans* in the cocultures. In addition, corresponding to the depletion of formate and acetate and a lower lactate concentration, the final pH of *C. cellulovorans*–*M. barkeri* (pH 6.7 ± 0.06) was higher than that of *C. cellulovorans*–*M. mazei* (pH 6.4 ± 0.05).

### Cell growth and culture morphology

**Fig. 4:**
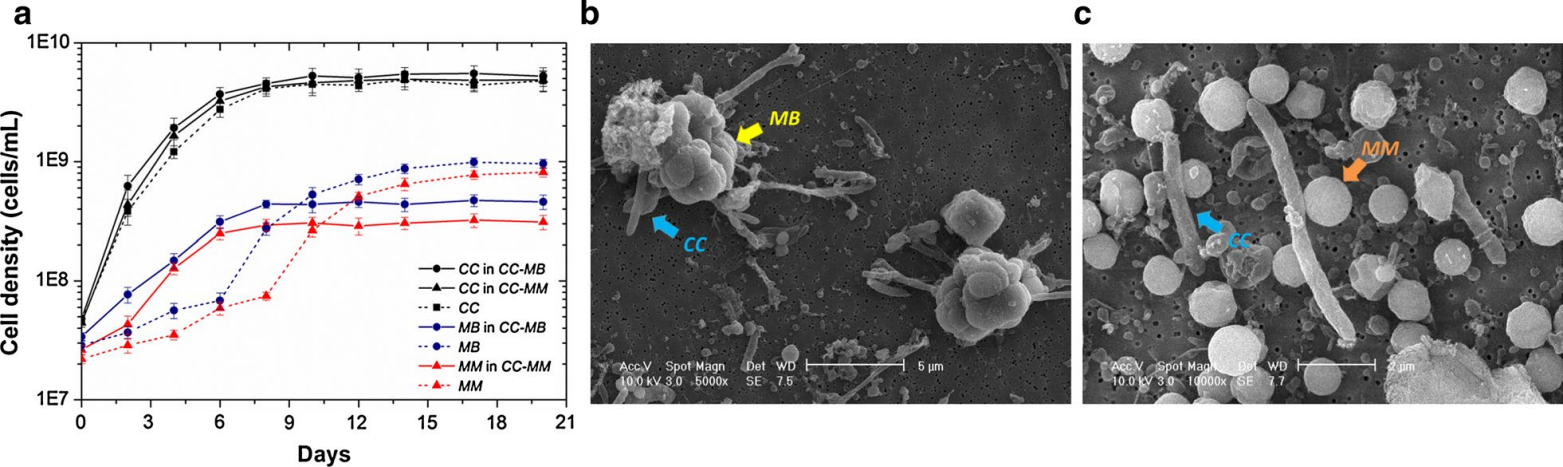
**a** Comparison of cell growth for cocultures of *C. cellulovorans*–*M. barkeri* (*CC*-*MB*) and *C. cellulovorans*–*M. mazei* (*CC*-*MM*) and monocultures of *C. cellulovorans* (*CC*), *M. barkeri* (*MB*), and *M. mazei* (*MM*). Each data point is an average of biological triplicate and *error bars* represent one standard deviation. Scanning electron microscopy image of the **b** *CC*–*MB* coculture and **c** *CC*–*MM* coculture

Cell growth of *M. barkeri* and *M. mazei* in cocultures was quantified by qPCR (Fig. 4a), and cell morphology was visualized by SEM (Fig. 4b, c). In cocultures, *M. barkeri* and *M. mazei* showed steady growth, reaching the stationary phase by day 8 (Fig. 4a). Compared to the monocultures of *M. barkeri* and *M. mazei* grown on acetate, *M. barkeri* and *M. mazei* in cocultures grew faster with a shorter lag phase. The cell yields of *M. barkeri* and *M. mazei* in cocultures were 3.1 × 10^13^ ± 4.5 × 10^12^ cells/mol of CH_4_ and 4.3 × 10^13^ ± 7.9 × 10^12^ cells/mol of CH_4_ respectively, whereas *M. barkeri* and *M. mazei* in monocultures were 3.5 × 10^13^ ± 3.2 × 10^12^ cells/mol of CH_4_ and 3.3 × 10^13^ ± 3.3 × 10^12^ cells/mol of CH_4_, respectively. *C. cellulovorans* in both monocultures and cocultures grew to a higher final cell concentration compared to the methanogens. However, unlike the different cell growth rates observed for the methanogens between monocultures and cocultures, the presence of methanogens did not result in significant differences in cell growth for *C. cellulovorans* between monocultures and cocultures (Fig. 4a). The *C. cellulovorans* growth yield in monocultures was 2.0 × 10^12^ ± 8.0 × 10^11^ cells/g of cellulose, whereas in cocultures of *C. cellulovorans*–*M. barkeri* and *C. cellulovorans*–*M. mazei,* it was 2.0 × 10^12^ ± 7.2 × 10^11^ cells/g of cellulose and 1.9 × 10^12^ ± 6.4 × 10^11^ cells/g of cellulose respectively. Interestingly, when grown in cocultures, *C. cellulovorans* and *M. barkeri* were observed to form aggregates, whereas *C. cellulovorans* and *M. mazei* existed as distinct separate cells (Fig. 4b, c).

### Expression of genes in pathways

**Fig. 5:**
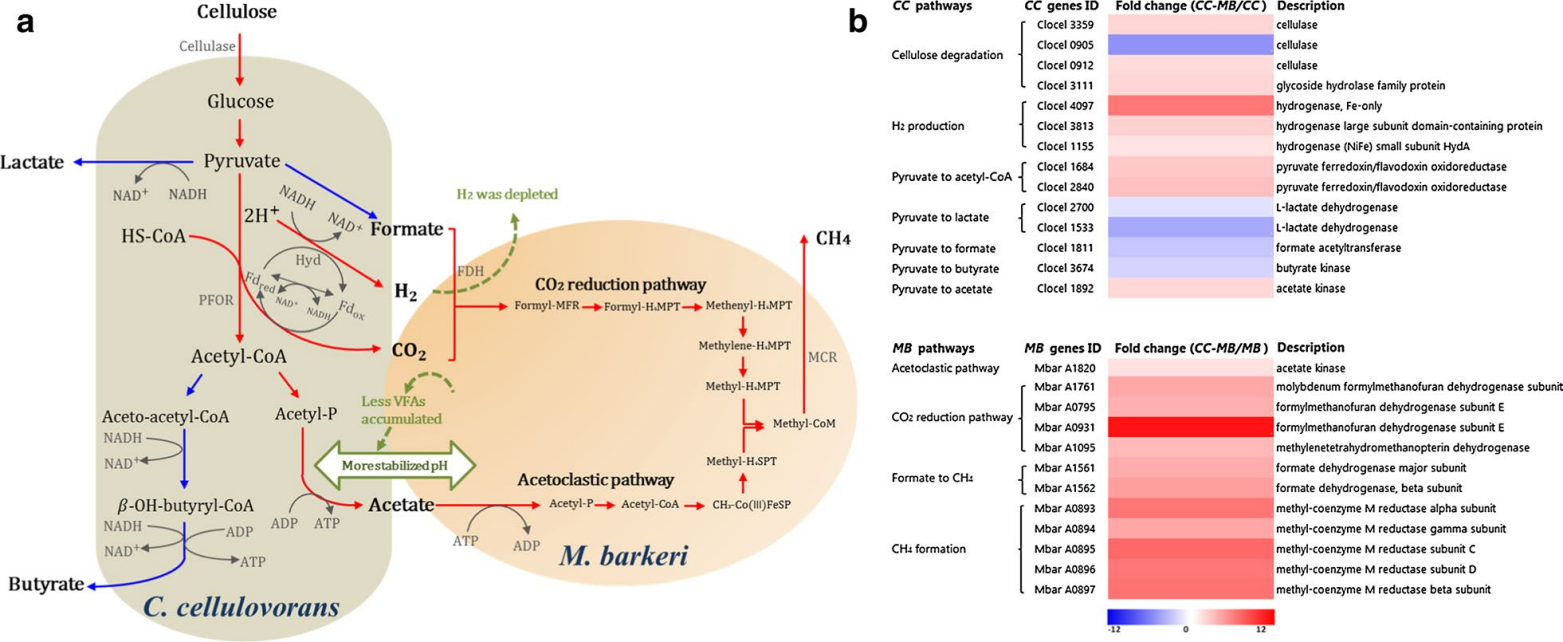
**a** A conceptual model illustrating the interactions between *C. cellulovorans* (*CC*) and *M. barkeri* (*MB*) in cocultures. The key pathways of the interactions are highlighted. The final products of the metabolic pathways are also shown in order to describe the carbon and cellular metabolism in the coculture model. *Red* and *blue color lines* represent the upregulated and downregulated pathways, respectively. *Green color* highlights the resulting effects of cocultivation of *CC* with *MB*. **b** Upregulated (*red*) and downregulated (*blue*) expression profiles of selected genes (|fold change| ≥1.2 and *p* value <0.05) of *CC* and *MB* related to the key pathways of the interactions at mid-exponential growth phase. The abbreviations are NADH, reduced form of nicotinamide adenine dinucleotide; NAD^+^, oxidized form of nicotinamide adenine dinucleotide; Hyd, hydrogenase; Fd_rex_, reduced form of ferredoxin; Fd_ox_, oxidized form of ferredoxin; PFOR, pyruvate:ferredoxin oxidoreductase; Acetyl-CoA, acetyl coenzyme A; Acetyl-P, acetyl phosphate; ATP, adenosine triphosphate; ADP, adenosine diphosphate; HS-CoA, coenzyme A; FDH, formate dehydrogenase; Formyl-MFR, a formyl-methanofuran; Formyl-H_4_MPT, 5-formyl-tetrahydromethanopterin; Methenyl-H_4_MPT, 5,10-methenyltetrahydromethanopterin; Methylene-H_4_MPT, 5,10-methylene-tetrahydromethanopterin; Methyl-H_4_MPT, 5-methyl-tetrahydromethanopterin; Methyl-H_4_SPT, 5-methyl-tetrahydrosarcinapterin; CH_3_-Co(III)FeSP, a methylated corrinoid Fe-S protein; MCR, methyl-coenzyme M reductase

**Fig. 6:**
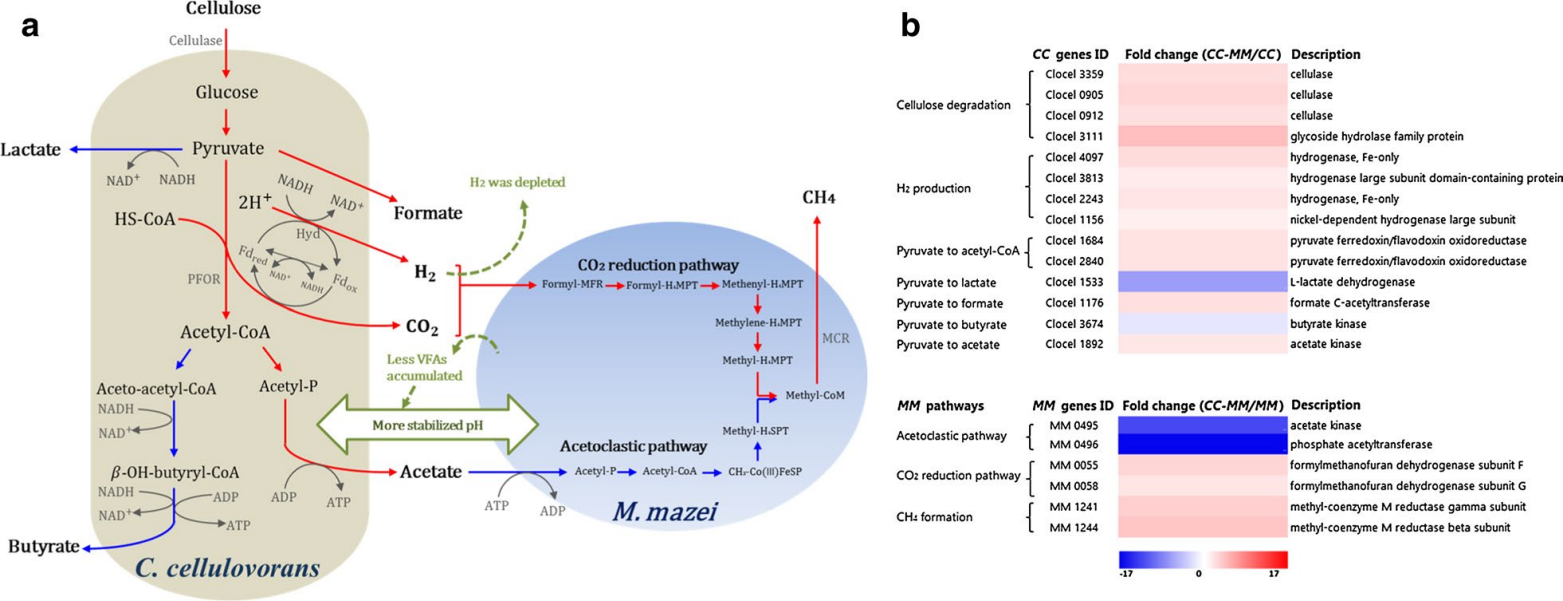
**a** A conceptual model illustrating the interactions between *C. cellulovorans* (*CC*) and *M. mazei* (*MM*) in cocultures. The key pathways of the interactions are *highlighted*. The final products of the metabolic pathways are also shown in order to describe the carbon and cellular metabolism in the coculture model. *Red* and *blue color lines* represent the upregulated and downregulated pathways, respectively. *Green color* highlights the resulting effects of cocultivation of *CC* with *MM*. **b** Upregulated (*red*) and downregulated (*blue*) expression profiles of selected genes (|fold change| ≥1.2 and *p* value <0.05) of *CC* and *MM* related to the key pathways of the interactions at mid-exponential growth phase. The abbreviations are NADH, reduced form of nicotinamide adenine dinucleotide; NAD^+^, oxidized form of nicotinamide adenine dinucleotide; Hyd, hydrogenase; Fd_rex_, reduced form of ferredoxin; Fd_ox_, oxidized form of ferredoxin; PFOR, pyruvate:ferredoxin oxidoreductase; Acetyl-CoA, acetyl coenzyme A; Acetyl-P, acetyl phosphate; ATP, adenosine triphosphate; ADP, adenosine diphosphate; HS-CoA, coenzyme A; Formyl-MFR, a formyl-methanofuran; Formyl-H_4_MPT, 5-formyl-tetrahydromethanopterin; Methenyl-H_4_MPT, 5,10-methenyltetrahydromethanopterin; Methylene-H_4_MPT, 5,10-methylene-tetrahydromethanopterin; Methyl-H_4_MPT, 5-methyl-tetrahydromethanopterin; Methyl-H_4_SPT, 5-methyl-tetrahydrosarcinapterin; CH_3_-Co(III)FeSP, a methylated corrinoid Fe-S protein; MCR, methyl-coenzyme M reductase

Based on the physiological results of the cocultures, a number of pathways that are important to the regulation and metabolism of the interactions between *C. cellulovorans* and *M. barkeri* or *C. cellulovorans* and *M. mazei* were investigated in detail (Figs. 5a, 6a). In order to examine how cells regulate these pathways, 17 *C. cellulovorans* genes, 13 *M. barkeri* genes, and 7 *M. mazei* genes encoding key enzymes within the respective pathways were analyzed by RT-qPCR. The fold changes of cocultures relative to their respective monocultures for these 37 selected genes are shown in Additional file 1: Tables S4, S5, S6. Genes with an absolute value of fold change ≥1.2 and a *p* value <0.05 were considered as differentially expressed to infer the regulation of the pathways (Figs. 5b, 6b).

For *C. cellulovorans* in the *C. cellulovorans*–*M. barkeri* cocultures (Fig. 5a, b), two out of three genes encoding cellulase (Clocel 3359 and Clocel 0912), the gene encoding glycoside hydrolase (Clocel 3111) related to cellulose degradation, and the genes encoding hydrogenase (Hyd) (Clocel 4097, Clocel 3813, and Clocel 1155) related to H_2_ production were all upregulated. The two genes (Clocel 1684 and Clocel 2840) encoding the pyruvate:ferredoxin oxidoreductase (PFOR) within the pathway of pyruvate to acetyl coenzyme A (acetyl-CoA) were upregulated. For VFA biosynthesis, the gene (Clocel 1892) encoding acetate kinase for catalyzing pyruvate to acetate was upregulated, whereas the gene (Clocel 3674) encoding butyrate kinase within the pathway of pyruvate to butyrate, the gene (Clocel 1811) encoding formate acetyltransferase within the pathway of pyruvate to formate, and the genes (Clocel 2700 and Clocel 1533) encoding l-lactate dehydrogenase within the pathway of pyruvate to lactate were all downregulated.

For *M. barkeri* in the cocultures, the gene (Mbar A1820) encoding acetate kinase within the acetoclastic pathway, the genes (Mbar A1761, Mbar A0795, Mbar A0931, and Mbar A1095) encoding formylmethanofuran dehydrogenase and methylene-tetrahydromethanopterin dehydrogenase within the CO_2_ reduction pathway, the genes (Mbar A0893–0897) encoding the methyl-coenzyme M reductase (MCR)—which catalyzes the final step in the formation of CH_4_—and the genes (Mbar A1561–1562) encoding formate dehydrogenase (FDH) were all upregulated.

For *C. cellulovorans* in the *C. cellulovorans*–*M. mazei* cocultures (Fig. 6a, b), the genes encoding cellulase (Clocel 3359, Clocel 0905, and Clocel 0912), glycoside hydrolase (Clocel 3111), and Hyd (Clocel 4097, Clocel 3813, Clocel 2243, and Clocel 1156) were all upregulated. The two genes (Clocel 1684 and Clocel 2840) encoding the PFOR were also upregulated. For VFA biosynthesis, the gene (Clocel 1892) encoding acetate kinase and the gene (Clocel 1176) encoding formate acetyltransferase were upregulated, whereas the gene (Clocel 3674) encoding butyrate kinase and the gene (Clocel 1533) encoding l-lactate dehydrogenase were downregulated in the *C. cellulovorans*–*M. mazei* cocultures.

For *M. mazei* in the cocultures, the genes (MM 0495–0496) encoding acetate kinase and phosphate acetyltransferase within the acetoclastic pathway were downregulated, and the genes (MM 0055 and MM 0058) encoding enzymes of the CO_2_ reduction pathway and the genes (MM 1241 and MM 1244) encoding the MCR were upregulated.

## Discussion

### Coculturing *C. cellulovorans* with *M. barkeri* or *M. mazei* enabled direct conversion of cellulose to CH_4_

Previous studies [25, 36] have shown that the cellulolytic bacterium *C. cellulovorans* is capable of fermenting cellulose to H_2_, CO_2_, and VFAs—including formate, acetate, butyrate, and lactate as the major metabolites—whereas genomic and physiological studies [21, 37–39] have demonstrated that *M. barkeri* and *M. mazei* possess the ability to couple growth and CH_4_ generation by the acetoclastic and the CO_2_ reduction pathways. Although *M. barkeri* has never been reported to utilize formate for growth in pure cultures, genomic studies [21] have revealed the presence of genes (Mbar A1561–1562) encoding the FDH, suggesting that *M. barkeri* has the potential to utilize formate for methanogenesis. In this study, coculturing *C. cellulovorans* with either *M. barkeri* or *M. mazei* both enabled CH_4_ production from cellulose and enhanced cellulose degradation compared to monocultures of *C. cellulovorans*. Surprisingly, in addition to H_2_ and acetate consumption, formate was also consumed in the *C. cellulovorans*–*M. barkeri* cocultures. This demonstrates that *M. barkeri* is able to simultaneously utilize the three precursors (H_2_, formate, and acetate) for methanogenesis. Consistent with previous results [21], we observed no growth of *M. barkeri* in monocultures when grown with 50 mM of formate alone after 9 months. Moreover, we mimicked the conditions of the *C. cellulovorans*–*M. barkeri* cocultures by artificially providing monocultures of *M. barkeri* with all three methanogenesis precursors (10 mM of formate, 3 mM of acetate, and 0.95 mmol of H_2_). However, without the presence of *C. cellulovorans,* neither formate nor acetate consumption was significant (only 1.5 ± 0.4 mM of formate, which is less than 15% of the total amount provided, and insignificant amount of acetate were consumed), though 0.95 mmol of H_2_ was depleted gradually during the cultivation period (no H_2_ accumulation was measured in the cocultures) (Additional file 1: Figure S2). The insignificant consumption of acetate might be due to the presence of the added H_2_ since the 0.95 mmol of added H_2_ was not exhausted immediately. An inhibitory effect of H_2_ on the acetoclastic methanogenesis in *Methanosarcina* spp. has previously been reported [40]. Although the cumulative CH_4_ production of *M. barkeri* monocultures grown with the three methanogenesis precursors was higher than *M. barkeri* monocultures grown with acetate and H_2_ (0.3 ± 0.01 mmol versus 0.2 ± 0.01 mmol) (Additional file 1: Figures S2, S3), it is significantly less than the *C. cellulovorans*–*M. barkeri* cocultures. The increase in cell density of the *M. barkeri* monocultures grown with the three methanogenesis precursors over the course of the experiment is also significantly lower than the *M. barkeri* in the *C. cellulovorans*–*M. barkeri* cocultures (7.5 × 10^7^ ± 2.9 × 10^7^ cells/mL versus 4.3 × 10^8^ ± 6.3 × 10^7^ cells/mL) (Additional file 1: Figure S4). These results indicate that monocultures of *M. barkeri* did not grow as robustly as the *M. barkeri* in the cocultures even though similar methanogenesis precursors were provided, suggesting the interactions between *C. cellulovorans* and *M. barkeri* might have provided additional metabolic benefits to *M. barkeri* besides supplying the three methanogenesis precursors. The observed physiology of *M. barkeri* in cocultures is reproducible, but whether or not a specific cellulolytic bacterium has to be the coculture partner remains to be determined.

According to the physiological characteristics of the cocultures, a conceptual model that describes the interactions between *C. cellulovorans* and *M. barkeri* or *M. mazei* has been developed (Figs. 5a, 6a). In the *C. cellulovorans*–*M. barkeri* cocultures, cellulose is first fermented to H_2_, formate, acetate, butyrate, and lactate by *C. cellulovorans* (Fig. 5a). *M. barkeri* then consumes the acetate, H_2_, and formate for cell growth and methanogenesis through both the acetoclastic (Eq. 1) and the CO_2_ reduction pathways with electrons derived from H_2_ and formate (Eqs. 2 and 3). The dependence of *M. barkeri* on the metabolites of *C. cellulovorans* is supported by the H_2_, VFAs, and pH data, which showed no H_2_ accumulation, transient accumulation of formate and acetate, and a more stabilized pH in the cocultures compared to monocultures of *C. cellulovorans*. Consistent with these physiological observations, the CO_2_ reduction pathway of *M. barkeri* was upregulated (Fig. 5b), supporting that H_2_ and CO_2_ were utilized for CH_4_ production in the *C. cellulovorans*–*M. barkeri* cocultures. Upregulation of the genes encoding the FDH further confirmed that formate was also used as an electron donor for methanogenesis through the CO_2_ reduction pathway. Unexpectedly, the acetoclastic pathway of *M. barkeri* was upregulated, suggesting that the CH_4_ production from acetate in the cocultures was more active than in the *M. barkeri* monocultures grown on acetate. Furthermore, upregulation of the genes encoding the MCR suggests that overall CH_4_ formation was enhanced in the *C. cellulovorans*–*M. barkeri* cocultures relative to the *M. barkeri* monocultures.

1 $${\text{CH}}_{ 3} {\text{COOH}} \to {\text{CH}}_{ 4} + {\text{CO}}_{ 2}$$

2 $$4 {\text{H}}_{ 2} + {\text{CO}}_{ 2} \to {\text{CH}}_{ 4} + 2 {\text{H}}_{ 2} {\text{O}}$$

3 $$4 {\text{HCOOH}} \to {\text{CH}}_{ 4} + 2 {\text{H}}_{ 2} {\text{O}} + 3 {\text{CO}}_{ 2}$$

In the *C. cellulovorans*–*M. mazei* cocultures (Fig. 6a), the interactions between *C. cellulovorans* and *M. mazei* are similar to those in the *C. cellulovorans*–*M. barkeri* cocultures, except that formate cannot be utilized by *M. mazei* for methanogenesis because the genes encoding FDH are absent in its genome [21, 22]. Previous studies [40, 41] have demonstrated that *M. mazei* can utilize H_2_ and acetate for methanogenesis. Our results showed no H_2_ was accumulated throughout the cultivation period in the *C. cellulovorans*–*M. mazei* cocultures, indicating that the H_2_ produced by *C. cellulovorans* was completely utilized for methanogenesis. The upregulation of the genes encoding formylmethanofuran dehydrogenase subunit F and G within the CO_2_ reduction pathway further confirmed that H_2_ was utilized for methanogenesis (Fig. 6b). For the acetoclastic pathway, as opposed to the depletion of acetate observed in the *C. cellulovorans*–*M. barkeri* cocultures, acetate accumulated to a higher concentration in the *C. cellulovorans*–*M. mazei* cocultures than in the *C. cellulovorans* monocultures. Moreover, the acetoclastic pathway of *M. mazei* was downregulated, suggesting that CH_4_ production from acetate in the cocultures is less active compared to the *M. mazei* monocultures grown on acetate. However, upregulation of the genes encoding the MCR of *M. mazei* suggested that overall CH_4_ formation is elevated in the *C. cellulovorans*–*M. mazei* cocultures.

### Stabilization of pH by methanogens enhanced cellulose degradation

Due to the consumption of formate and acetate by *M. barkeri* and the reduction in lactate and butyrate production by *C. cellulovorans*, a more stabilized pH was observed in the *C. cellulovorans*–*M. barkeri* cocultures than in the *C. cellulovorans* monocultures. Studies [35] have shown that acidic pH can inhibit both the H_2_ production and the metabolic activity of dark fermenters. Therefore, the more stabilized pH could potentially improve the cellular metabolism of *C. cellulovorans* in the cocultures. Accordingly, our results showed that both the rate and extent of cellulose degradation were enhanced (Table 1), and the cellulose degradation pathway of *C. cellulovorans* was upregulated in the *C. cellulovorans*–*M. barkeri* cocultures (Fig. 5b). As a result of a lower accumulated concentration of VFAs, the pH of the *C. cellulovorans*–*M. mazei* cocultures was also more stabilized than that of the *C. cellulovorans* monocultures. However, the pH of the *C. cellulovorans*–*M. mazei* cocultures was lower than that of the *C. cellulovorans*–*M. barkeri* cocultures as formate and acetate both accumulated in the *C. cellulovorans*–*M. mazei* cocultures. Cellulose degradation was also enhanced in the *C. cellulovorans*–*M. mazei* cocultures compared to the *C. cellulovorans* monocultures, but to a smaller extent compared to the *C. cellulovorans*–*M. barkeri* cocultures. Correspondingly, the cellulose degradation pathway of *C. cellulovorans* was also upregulated in the *C. cellulovorans*–*M. mazei* cocultures (Fig. 6b). However, the additional cellulose degraded, and the more optimal pH did not lead to a significant increase in *C. cellulovorans* cell density in either coculture.

### Simultaneous utilization of three methanogenesis precursors promoted CH_4_ yield

The CH_4_ yield of the *C. cellulovorans*–*M. barkeri* cocultures (0.87 ± 0.02 mol CH_4_/mol glucose equivalent) was substantially higher than that of the *C. cellulovorans*–*M. mazei* cocultures (0.44 ± 0.04 mol CH_4_/mol glucose equivalent) and this could be attributed to two causes. First, besides H_2_ and acetate, additional electrons can be derived from formate for methanogenesis in the *C. cellulovorans*–*M. barkeri* cocultures, whereas only H_2_ and acetate can be utilized in the *C. cellulovorans*–*M. mazei* cocultures. Second, compared to the *C. cellulovorans*–*M. mazei* cocultures, the more stabilized pH of the *C. cellulovorans*–*M. barkeri* cocultures enabled cellulose degradation to be more complete, which resulted in additional H_2_, formate, and acetate being produced and provided additional substrates for CH_4_ production. The simultaneous utilization of H_2_, formate, and acetate for methanogenesis in the *C. cellulovorans*–*M. barkeri* cocultures also has advantages over other reported coculture models, in which only H_2_ and formate can be utilized for CH_4_ production. For example, Celine et al. [17] incorporated the H_2_-consuming methanogens from the human colon with the H_2_-producing fibrolytic strains to produce CH_4_ from cellulose, obtaining the highest CH_4_ yield of 0.33 ± 0.037 mol CH_4_/mol glucose equivalent. Bauchop et al. [19] employed the rumen H_2_- and formate-utilizing methanogens and the rumen anaerobic fungus for methanogenesis from cellulose, achieving the highest CH_4_ yield of 0.59 ± 0.009 mol CH_4_/mol glucose equivalent. Both of these CH_4_ yields are significantly lower than the yield obtained in the *C. cellulovorans*–*M. barkeri* cocultures (Table 1).

### Metabolic shifts in *C. cellulovorans* in cocultures

According to the stoichiometric Eqs. 1 through 3, a theoretical maximum CH_4_ production of 0.9 mmol is expected from the acetoclastic and CO_2_ reduction pathways based on 0.3 ± 0.02 mmol of acetate, 1.5 ± 0.08 mmol of H_2_, and 0.9 ± 0.04 mmol of formate produced in the monocultures of *C. cellulovorans*. However, in the *C. cellulovorans*–*M. barkeri* cocultures, a final CH_4_ amount of 1.5 ± 0.07 mmol was measured, which exceeds the theoretical maximum production by 66% according to the *C. cellulovorans* monocultures. This suggests that the sum of H_2_, formate, and acetate produced by *C. cellulovorans* in the *C. cellulovorans*–*M. barkeri* cocultures exceeds that of the *C. cellulovorans* monocultures.

Because the concentrations of H_2_, formate, and acetate measured in the cocultures were determined by both the production rate of *C. cellulovorans* and the consumption rate of *M. barkeri*, it is difficult to accurately calculate the concentration of each metabolite produced. In fact, no H_2_ accumulation was measured in the cocultures. Alternatively, the gene expressions of the H_2_, formate, and acetate production pathways were examined. Upregulation of the PFOR and Hyd in the cocultures (Fig. 5b) supports that H_2_ production of *C. cellulovorans* in the *C. cellulovorans*–*M. barkeri* cocultures was more active relative to the *C. cellulovorans* monocultures. Concomitant with the enhanced H_2_ formation, acetate production and the associated adenosine triphosphate (ATP) synthesis for *C. cellulovorans* might also be elevated as additional acetyl-CoA is generated from the enhanced oxidative decarboxylation of pyruvate. Correspondingly, upregulation of the pyruvate to acetate pathway in the cocultures was observed (Fig. 5b). In contrast, downregulation of the pyruvate to formate pathway suggests that the level of formate production was lower in the cocultures (Fig. 5b). On the other hand, the final concentrations of lactate and butyrate in the *C. cellulovorans*–*M. barkeri* cocultures were lower than in the *C. cellulovorans* monocultures. Correspondingly, the pathways of pyruvate to lactate and butyrate were both downregulated. Based on the gene expression, the increase in H_2_ and acetate production together with the decrease in formate, lactate, and butyrate production show that the fermentation pattern of *C. cellulovorans* in the *C. cellulovorans*–*M. barkeri* cocultures shifted relative to the *C. cellulovorans* monocultures.

In the *C. cellulovorans*–*M. mazei* cocultures, because acetate accumulated, it is difficult to judge whether acetate was actually consumed for methanogenesis. However, the final produced CH_4_ was 0.7 ± 0.09 mmol in the *C. cellulovorans*–*M. mazei* cocultures, which is 0.05 mmol more than the theoretical maximum CH_4_ yield expected from the sum of H_2_ (1.5 ± 0.08 mmol) and acetate (0.3 ± 0.02 mmol) produced by *C. cellulovorans* in the monocultures. This suggests that the sum of H_2_ and acetate consumed by *M. mazei* in the cocultures is more than that produced by *C. cellulovorans* in the monocultures. If acetate was not consumed and excluded for the methanogenesis, the measured 0.7 ± 0.09 mmol of CH_4_ production in the *C. cellulovorans*–*M. mazei* cocultures would require at least 2.9 mmol of H_2_ (93% higher than the H_2_ produced in the *C. cellulovorans* monocultures) from *C. cellulovorans* based on the stoichiometric Eq. 2. Instead of such a dramatic increase in H_2_ production in cocultures, it is more reasonable to postulate that acetate was consumed and also contributed to the total CH_4_ production in the *C. cellulovorans*–*M. mazei* cocultures. If this is the case, the actual acetate produced by *C. cellulovorans* in the cocultures was significantly more than that produced in the monocultures because there was still 0.4 ± 0.02 mmol of acetate left in the cocultures. The expected higher H_2_ and acetate production based on the stoichiometric calculation is supported by the upregulation of the H_2_ and acetate production pathways of *C. cellulovorans* in the *C. cellulovorans*–*M. mazei* cocultures. The final concentrations of lactate and butyrate in the *C. cellulovorans*–*M. mazei* cocultures were lower than in the *C. cellulovorans* monocultures, and the pathways of pyruvate to lactate and butyrate correspondingly were both downregulated (Fig. 6b). However, inconsistent with the upregulation of the pyruvate to formate pathway, the final concentration of formate in the *C. cellulovorans*–*M. mazei* cocultures was lower than that of the *C. cellulovorans* monocultures (Fig. 6b). Overall, these results suggest that the interactions between *C. cellulovorans* and *M. mazei* also induced a metabolic shift in the fermentation pattern of *C. cellulovorans*.

## Conclusions

In this study, we report a physiological and molecular investigation of two artificially constructed cocultures utilizing cellulose as the sole carbon substrate. In the cocultures of *C. cellulovorans*–*M. barkeri*, whereas *C. cellulovorans* produced H_2_, formate, acetate, butyrate, and lactate as the obligatory fermentation products from cellulose degradation, *M. barkeri* was able to further utilize H_2_, formate, and acetate for methanogenesis by both the CO_2_ reduction and acetoclastic pathways. Similar interactions were also observed in the *C. cellulovorans*–*M. mazei* cocultures, except that formate cannot be utilized by *M. mazei* for CH_4_ production. A shift in the fermentation pattern in *C. cellulovorans* was observed in both cocultures and the more stabilized pH promoted cellulose degradation and CH_4_ production. This study illustrates that the use of a constructed coculture to convert cellulosic biomass to CH_4_ is a viable strategy to produce renewable energy, and the interactions between the microbial partners could lead to beneficial outcomes. Understanding the microbial interactions in such an artificial coculture could provide fundamental guidance in engineering synthetic consortia for a more efficient large-scale biomethanation process.

## Additional file

**Additional file 1:Table S1.** Primer sequences for selected genes in RT-qPCR. **Table S2.** Primer sequences for internal control genes in RT-qPCR. **Figure S1.** Acetate concentration and CH_4_ production profiles of (a) monocultures of *M. barkeri* and (b) monocultures of *M. mazei*. **Figure S2.** (a) Acetate and formate concentrations and (b) H_2_ consumption and CH_4_ production of monocultures of *M. barkeri* grown with 10 mM of formate, 3 mM of acetate, and 0.95 mmol of H_2_. **Figure S3.** (a) Acetate concentration and (b) H_2_ consumption and CH_4_ production of monocultures of *M. barkeri* grown with 3 mM of acetate and 0.95 mmol of H_2_. **Table S3.** Products of cellulose fermentation by *C. cellulovorans* monocultures. **Table S4.** Gene expression of the selected *C. cellulovorans* (*CC*) genes in response to cocultivation with *M. barkeri* (*MB*) or *M. mazei* (*MM*) at mid-exponential growth phase. **Table S5.** Gene expression of the selected *M. barkeri* (*MB*) genes in response to cocultivation with *C. cellulovorans* (*CC*) at mid-exponential growth phase. **Table S6.** Gene expression of the selected *M. mazei* (*MM*) genes in response to cocultivation with *C. cellulovorans* (*CC*) at mid-exponential growth phase **Figure S4.** Comparison of the increase in cell density over the course of the experiment. Cell density at time zero was about 3.0 × 10^7^ cells/mL for the experiments.

## Abbreviations

CH_4_: methane
H_2_: hydrogen
CO_2_: carbon dioxide
NADH: nicotinamide adenine dinucleotide
NAD^+^: oxidized form of nicotinamide adenine dinucleotide
SEM: scanning electron microscopy
qPCR: quantitative PCR
RIN: RNA integrity number
RT: reverse transcription
cDNA: complementary DNA
Ct: comparative threshold
*gap*: glyceraldehyde dehydrogenase
VFAs: volatile fatty acids
Hyd: hydrogenase
PFOR: pyruvate:ferredoxin oxidoreductase
acetyl-CoA: acetyl coenzyme A
acetyl-P: acetyl phosphate
MCR: methyl-coenzyme M reductase
FDH: formate dehydrogenase
ATP: adenosine triphosphate
ADP: adenosine diphosphate
FADH_2_: flavin-adenine dinucleotide
Fd_rex_: reduced form of ferredoxin
Fd_ox_: oxidized form of ferredoxin
HS-CoA: coenzyme A
Formyl-MFR: a formylmethanofuran
Formyl-H_4_MPT: 5-formyl-tetrahydromethanopterin
Methenyl-H_4_MPT: 5,10-methenyltetrahydromethanopterin
Methylene-H_4_MPT: 5,10-methylene-tetrahydromethanopterin
Methyl-H_4_MPT: 5-methyl-tetrahydromethanopterin
Methyl-H_4_SPT: 5-methyl-tetrahydrosarcinapterin
CH_3_-Co(III)FeSP: a methylated corrinoid Fe-S protein
