# Supplementary material for: Physiological and molecular characterizations of the interactions in two cellulose-to-methane cocultures
Source: Biotechnol Biofuels. 2017 Feb 7;10:37. doi: 10.1186/s13068-017-0719-y (PMC5297212; doi:10.1186/s13068-017-0719-y)
Supplement: Supplementary file 1 — Additional file 1:Table S1. Primer sequences for selected genes in RT-qPCR. Table S2. Primer sequences for internal control genes in RT-qPCR. Figure S1. Acetate concentration and CH4 production profiles of (a) monocultures of M. barkeri and (b) monocultures of M. mazei. Figure S2. (a) Acetate and formate concentrations and (b) H2 consumption and CH4 production of monocultures of M. barkeri grown with 10 mM of formate, 3 mM of acetate, and 0.95 mmol of H2. Figure S3. (a) Acetate concentration and (b) H2 consumption and CH4 production of monocultures of M. barkeri grown with 3 mM of acetate and 0.95 mmol of H2. Table S3. Products of cellulose fermentation by C. cellulovorans monocultures. Table S4. Gene expression of the selected C. cellulovorans (CC) genes in response to cocultivation with M. barkeri (MB) or M. mazei (MM) at mid-exponential growth phase. Table S5. Gene expression of the selected M. barkeri (MB) genes in response to cocultivation with C. cellulovorans (CC) at mid-exponential growth phase. Table S6. Gene expression of the selected M. mazei (MM) genes in response to cocultivation with C. cellulovorans (CC) at mid-exponential growth phase Figure S4. Comparison of the increase in cell density over the course of the experiment. Cell density at time zero was about 3.0 × 10^7^ cells/mL for the experiments. [file 13068_2017_719_MOESM1_ESM.doc]

**Supplementary Materials**

**Physiological and molecular characterizations of the interactions in two cellulose-to-methane cocultures**

Hongyuan Lu, Siu-Kin Ng, Yangyang Jia, Mingwei Cai, and Patrick K. H. Lee^#^

School of Energy and Environment, City University of Hong Kong, Hong Kong

**Additional file 1:** Primer sequences for selected genes in RT-qPCR

| **Gene** | **Sequence (5’ to 3’)** |
| --- | --- |
| Clocel 1155 Fw. | GCTGTGGGAACTTGTGCTTCT |
| Clocel 1155 Rev. | TGCAGGACAGCCTGGAACTC |
|  |  |
| Clocel 1156 Fw. | CGCAAAGATAGATGCTGTTTCAA |
| Clocel 1156 Rev. | TGCCTTCCCGCCTAAGATT |
|  |  |
| Clocel 1176 Fw. | TTGGTAACGACGATCCAGAAATT |
| Clocel 1176 Rev. | TTCTTCGGCAGGTTCAGGTT |
|  |  |
| Clocel 1811 Fw. | TGGTGTTGCGGGTGTGTCT |
| Clocel 1811 Rev. | GGGAAGTCTCCTTCGATTTTGA |
|  |  |
| Clocel 1533 Fw. | CAGTGCTTCCAGTTGGAGCTTTA |
| Clocel 1533 Rev. | TCTCACGCCTTCCCAACCTA |
|  |  |
| Clocel 1684 Fw. | GGCGCAGTTCATGGCTCTT |
| Clocel 1684 Rev. | GCTCTAGCTGAAACGTGGAACA |
|  |  |
| Clocel 1892 Fw. | GGAGAAAATGCTGCACTGGTAA |
| Clocel 1892 Rev. | ACCAGCTGCAGCCTCATTAAG |
|  |  |
| Clocel 2243 Fw. | TGCTGCGGATTTGACCATAA |
| Clocel 2243 Rev. | CCATCCTGGACAGCAACTTGT |
|  |  |
| Clocel 2700 Fw. | ATCAGGGCTACCCAAAAATCAG |
| Clocel 2700 Rev. | TCTCCGTGTTCACCCATTGA |
|  |  |
| Clocel 2840 Fw. | TGGATGAGGCTCTCGGCTAT |
| Clocel 2840 Rev. | TGCATCAGCACCCTTGTCAA |
|  |  |
| Clocel 3674 Fw. | GTCATATGGGCGGAGGAGTTT |
| Clocel 3674 Rev. | AGTTCCGCTTCTTTCTGGAGAA |
|  |  |
| Clocel 3813 Fw. | TACAGATTGCCCTGATCCTTCA |
| Clocel 3813 Rev. | CAACCAGTGCAGGCATCATT |
|  |  |
| Clocel 0905 Fw. | CGTCTTTCAGCAGAGGCATCT |
| Clocel 0905 Rev. | CGTTGCAGGTGTCGCTGTAA |
|  |  |
| Clocel 3359 Fw. | ACAACAAAAGGCACTACGACACA |
| Clocel 3359 Rev. | CTAGTAGCGTCGATGGCATTGA |
|  |  |
| Clocel 3111 Fw. | ATGCCAACTGACCCAGCAA |
| Clocel 3111 Rev. | TCTTCAGCTGTTCCCCAGGTA |
|  |  |
| Clocel 0912 Fw. | ACAGCGCAAGATGGCTTCTA |
| Clocel 0912 Rev. | GCTGTAGCTCCCCATTGAGT |
|  |  |
| Clocel 4097 Fw. | ATCGGCGGAAGTGAGTATAACGT |
| Clocel 4097 Rev. | AGGTTGACCTCCTCCGTTCA |
|  |  |
| Mbar A0893 Fw. | ATGCAGCAGATGTGGGATGAC |
| Mbar A0893 rev. | CGACCATCATTTCCTGAACCA |
|  |  |
| Mbar A1821 Fw. | CGCTGTTATGGCGGCTAAA |
| Mbar A1821 Rev. | AGCAGATGCGAGAGCTGCAT |
|  |  |
| Mbar A1820 Fw. | GCTGCCTCTCATGGAAACGA |
| Mbar A1820 Rev. | CACCATTGAGCACGGCTAAA |
|  |  |
| Mbar A1761 Fw. | CTGGCGAAAATGCTGGAGAA |
| Mbar A1761 Rev. | AACATCGGCTCCCAGCTTAA |
|  |  |
| Mbar A0795 Fw. | CTGTCCCGGAATTTCCATTG |
| Mbar A0795 Rev. | CGCAGGACCGGTTTTCTACA |
|  |  |
| Mbar A0931 Fw. | CGGGACTGACCATCGGTTAT |
| Mbar A0931 Rev. | TCCGCATGCGTCATTTTCTA |
|  |  |
| Mbar A1095 Fw. | TCCGGAAAATCAGGAAAGGA |
| Mbar A1095 Rev. | GCGAAGCCTGCGTACTCAAC |
|  |  |
| Mbar A0894 Fw. | CCAGCGAAGAAGCTGCAAAG |
| Mbar A0894 Rev. | TTCGACGACTTCTGCATCGT |
|  |  |
| Mbar A0895 Fw. | TTTGCAGAGGCAGGGATAAAG |
| Mbar A0895 Rev. | TCCGCTCACCATATCCATCA |
|  |  |
| Mbar A0896 Fw. | AGATCTGTGTGGGCAGGATCA |
| Mbar A0896 Rev. | GGAATGGGAGCAATTTTTCG |
|  |  |
| Mbar A0897 Fw. | GGTCGATGAAGGCGACGATA |
| Mbar A0897 Rev. | CCAACTGTTGTTGCGGACAT |
|  |  |
| Mbar A1561 Fw | TCGGACCCGGATCTAAACAA |
| Mbar A1561 Rev | ATTGGTCTGGGTCCCGTTCT |
|  |  |
| Mbar A1562 Fw. | TGCAAAAGGCGCAGAACTACT |
| Mbar A1562 Rev. | GCCAGCTTCAGCATCGACTT |
|  |  |
| MM 0495 Fw. | CTGTCGGGCACAGAGTTGTG |
| MM 0495 Rev. | GCCAGTTCGAAGCAGTCCTT |
|  |  |
| MM 0496 Fw. | TTGCAGGCGATCTGGATCTC |
| MM 0496 Rev. | CTTCGAGTGTGACGCCTTTG |
|  |  |
| MM 0055 Fw. | CCGCGCAAGATAGCTTTTG |
| MM 0055 Rev. | TCGGTGTGGTGGACATCAGT |
|  |  |
| MM 0058 Fw. | GCCTGCCCTCCGAATGTAG |
| MM 0058 Rev. | AGGACAAGCCTGCCAGGATT |
|  |  |
| MM 0059 Fw. | AACACGGGCCGGAATATTCT |
| MM 0059 Rev. | CGGGTCCGAACCAATGATAA |
|  |  |
| MM 1241 Fw. | ACGTCCAGTTCGCTGACTCA |
| MM 1241 Rev. | TCACGGGCTTCAACAATCTG |
|  |  |
| MM 1244 Fw. | CGGTAAGGACGGAACCATTG |
| MM 1244 Rev. | CATTGGGACGTCGTTTGCTT |

**Additional file 2:** Primer sequences for internal control genes in RT-qPCR

| **Gene** | **Sequence (5’ to 3’)** |
| --- | --- |
| Mbar A2189 Fw. | CAATCGTACCCGACCCAATC |
| Mbar A2189 Rev. | CAGGGTTGTCGGGATCTTCA |
|  |  |
| Clocel 3871 Fw. | GGTGTAGGCGGTCCTGGATA |
| Clocel 3871 Rev. | CCAGCAGAATTTGGCATCATAG |
|  |  |
| MM 2782 Fw. | GAAGTCAACACCGACTGCAC |
| MM 2782 Rev. | GCCCCATGTCTCTTGCTAAT |

**
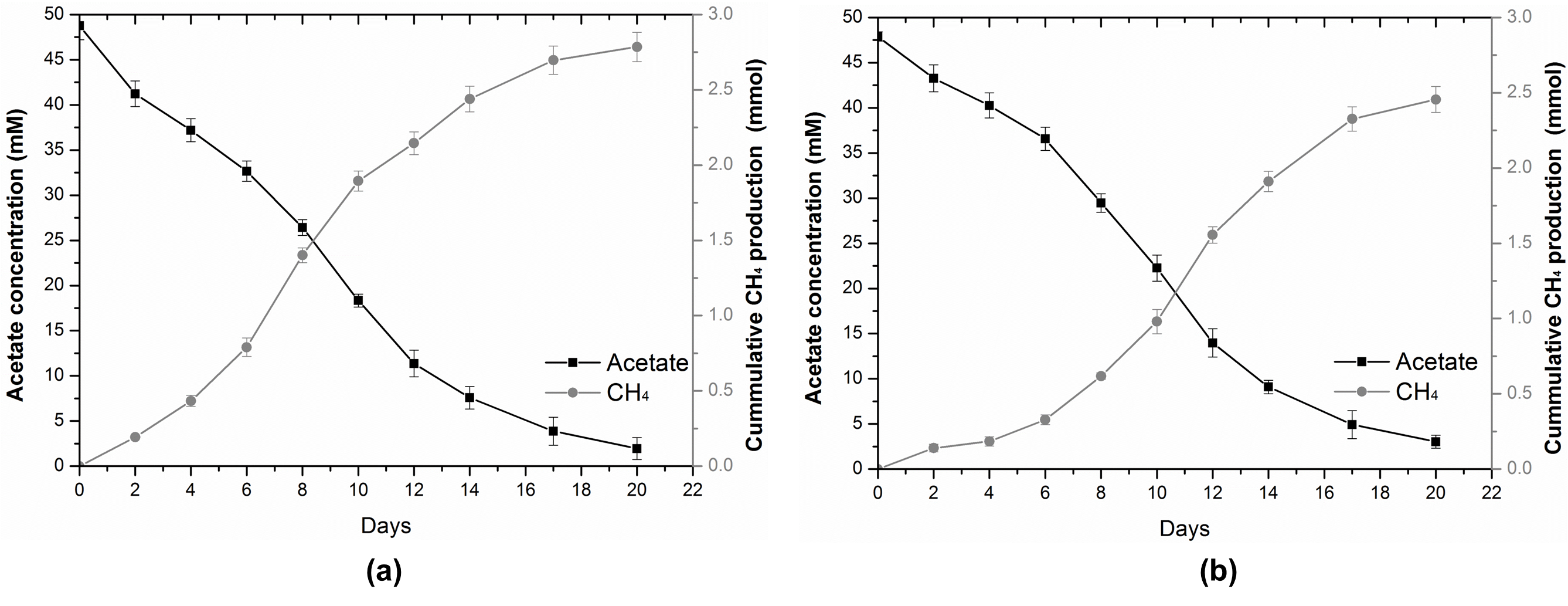
**

**Additional file 3:** Acetate concentration and CH_4_ production profiles of (a) monocultures of *M. barkeri* and (b) monocultures of *M. mazei*. Each data point is an average of biological triplicate and error bars represent one standard deviation.

**
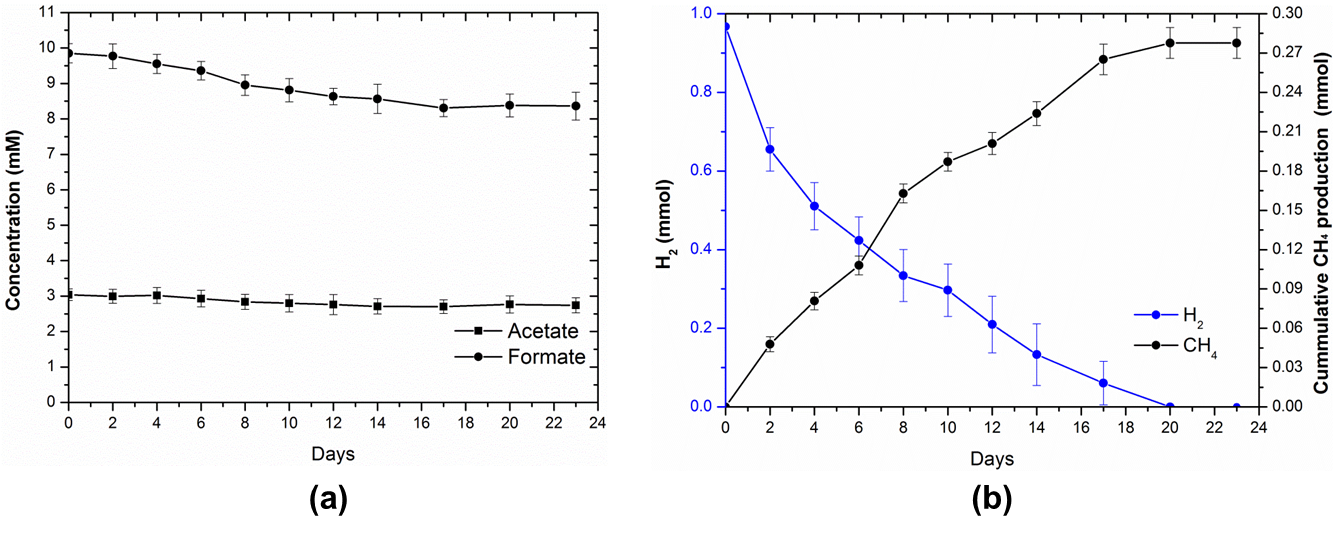
**

**Additional file 4:** (a) Acetate and formate concentrations and (b) H_2_ consumption and CH_4_ production of monocultures of *M. barkeri* grown with 10 mM of formate, 3 mM of acetate, and 0.95 mmol of H_2_. Each data point is an average of biological triplicate and error bars represent one standard deviation.

**
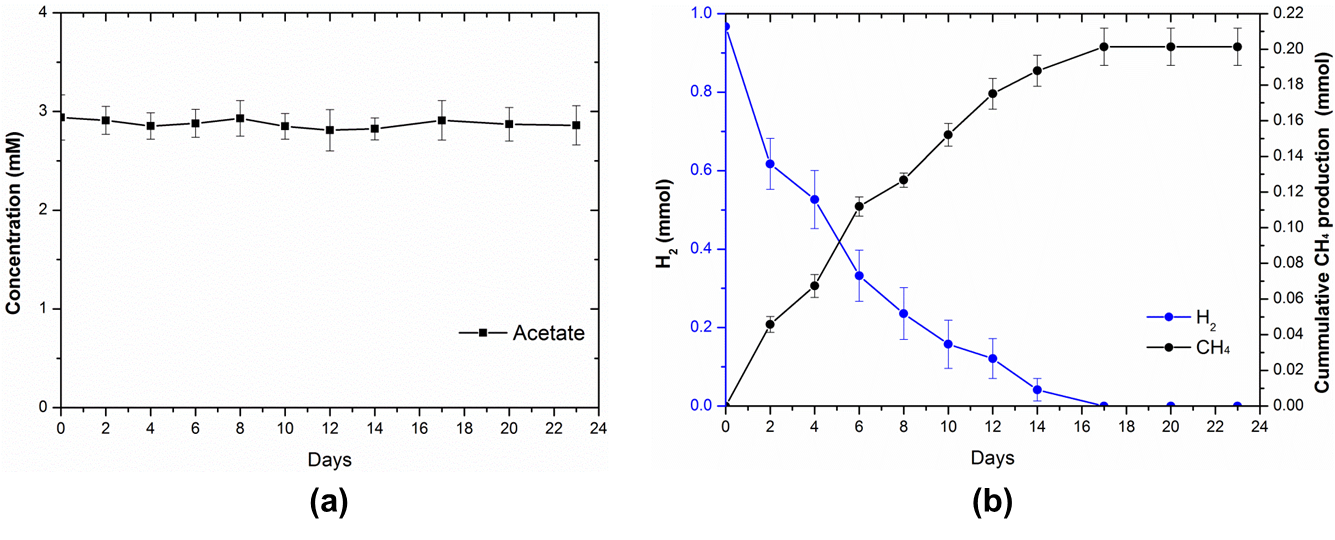
**

**Additional file 5:** (a) Acetate concentration and (b) H_2_ consumption and CH_4_ production of monocultures of *M. barkeri* grown with 3 mM of acetate and 0.95 mmol of H_2_. Each data point is an average of biological triplicate and error bars represent one standard deviation.

**Additional file 6:** Products of cellulose fermentation by *C. cellulovorans* monocultures

| **Product** | **Amount formed (mmol/g of cellulose)** |
| --- | --- |
| Acetate | 1.28 ± 0.07 |
| Butyrate | 3.84 ± 0.14 |
| Formate | 3.89 ± 0.15 |
| Lactate | 1.55 ± 0.05 |
| H_2_ | 6.54 ± 0.32 |
| CO_2_ | NA^*^ |
| Data are the average (± one standard derivation) of biological triplicate. ^*^ The CO_2_ concentration was not determined because excess CO_2_ was provided in the headspace of the culture bottles with CO_2_/N_2_ (20%:80%) and 2.5 g/L of sodium bicarbonate was added as part of the DCB-1 medium. | |

**Additional file 7: Gene expression of the selected *C.* *cellulovorans* (*CC*) genes in response to cocultivation with *M. barkeri* (*MB*) or *M. mazei* (*MM*) at mid-exponential growth phase**

| ***CC* pathway**  **and gene ID** | **Description** | **Fold-change**^†^ **(*CC-MB*/*CC*)** | ***p*-value**^*^ | **Fold-change**^†^ **(*CC-MM*/*CC*)** | ***p*-value**^*^ |
| --- | --- | --- | --- | --- | --- |
| **H_2_ production (5 genes)** | |  |  |  |  |
| Clocel 4097 | hydrogenase, Fe-only | 6.4 ± 0.2 | 4.1E-08 | 2.5 ± 0.3 | 2.0E-04 |
| Clocel 3813 | hydrogenase large subunit domain-containing protein | 2.3 ± 0.7 | 2.4E-03 | 1.5 ± 0.2 | 3.7E-02 |
| Clocel 2243 | hydrogenase, Fe-only | 1.2 ± 0.3 | 1.9E-01 | 1.9 ± 0.4 | 2.4E-04 |
| Clocel 1155 | hydrogenase (NiFe) small subunit HydA | 1.4 ± 0.3 | 4.4E-02 | 1.5 ± 0.3 | 9.4E-02 |
| Clocel 1156 | nickel-dependent hydrogenase large subunit | 1.1 ± 0.1 | 2.2E-01 | 1.3 ± 0.3 | 2.1E-02 |
| **Cellulose degradation (4 genes)** | |  |  |  |  |
| Clocel 3359 | cellulase, glycosyl hydrolases family 5 protein | 2.1 ± 0.3 | 3.5E-03 | 2.4 ± 0.3 | 9.1E-04 |
| Clocel 0905 | cellulase, glycosyl hydrolases family 5 protein | -5.2 ± 1.5 | 1.1E-04 | 2.9 ± 0.4 | 2.7E-03 |
| Clocel 0912 | cellulase, glycosyl hydrolases family 5 protein | 1.8 ± 0.3 | 4.0E-03 | 2.2 ± 0.3 | 1.2E-03 |
| Clocel 3111 | glycosyl hydrolases family 5 protein | 2.1 ± 0.5 | 2.1E-04 | 4.4 ± 1.0 | 1.4E-05 |
| **Pyrurate to acetyl coenzyme A (2 genes)** | |  |  |  |  |
| Clocel 1684 | pyruvate ferredoxin/flavodoxin oxidoreductase | 2.6 ± 0.4 | 2.9E-03 | 2.1 ± 0.1 | 1.4E-03 |
| Clocel 2840 | pyruvate ferredoxin/flavodoxin oxidoreductase | 3.1 ± 0.7 | 3.1E-03 | 2.1 ± 0.3 | 3.3E-03 |
| **Pyrurate to acetate (1 gene)** | | | | | |
| Clocel 1892 | acetate kinase | 2.0 ± 0.4 | 3.0E-03 | 1.8 ± 0.4 | 2.5E-04 |
| **Pyrurate to butyrate (1 gene)** | |  |  |  |  |
| Clocel 3674 | butyrate kinase | -2.1 ± 0.2 | 3.0E-04 | -1.8 ± 0.5 | 2.0E-02 |
| **Pyrurate to formate (2 genes)** | |  |  |  |  |
| Clocel 1176 | formate C-acetyltransferase | 1.1 ± 0.4 | 7.4E-01 | 2.2 ± 0.7 | 8.6E-03 |
| Clocel 1811 | formate acetyltransferase | -2.8 ± 1.1 | 7.0E-04 | 1.3 ± 0.3 | 9.3E-02 |
| **Pyrurate to latate (2 genes)** | |  |  |  |  |
| Clocel 2700 | L-lactate dehydrogenase | -1.4 ± 0.2 | 1.2E-02 | 0.6 ± 1.2 | 2.7E-01 |
| Clocel 1533 | L-lactate dehydrogenase | -4.2 ± 0.7 | 1.2E-05 | -6.6 ± 0.7 | 1.3E-06 |
| A gene with an absolute value of the fold-change ≥ 1.2^†^ and a *p-*value < 0.05^*^ is considered differentially expressed | | | |  |  |

***Additional file 8: Gene expression of the selected M. barkeri (MB) genes in response to cocultivation with C. cellulovorans (CC) at mid-exponential growth phase***

| ***MB* pathway**  **and gene ID** | **Description** | **Fold-change**^†^ **(*CC-MB*/*MB*)** | ***p*-value**^*^ |
| --- | --- | --- | --- |
| **Acetoclastic pathway (2 genes)** | |  |  |
| Mbar A1820 | acetate kinase | 1.5 ± 0.4 | 4.0E-02 |
| Mbar A 1821 | phosphate acetyltransferase | 1.5 ± 0.7 | 1.8E-01 |
| **CO_2_ reduction pathway (4 genes)** | |  |  |
| Mbar A 1761 | molybdenum formylmethanofuran dehydrogenase subunit | 4.2 ± 0.7 | 2.2E-05 |
| Mbar A 0795 | formylmethanofuran dehydrogenase subunit E | 3.8 ± 0.7 | 1.3E-04 |
| Mbar A 0931 | formylmethanofuran dehydrogenase subunit E | 11.1 ± 1.3 | 5.3E-06 |
| Mbar A 1095 | methylenetetrahydromethanopterin dehydrogenase | 3.4 ± 0.9 | 5.3E-05 |
| **CH_4_ formation (5 genes)** | |  |  |
| Mbar A 0893 | methyl-coenzyme M reductase alpha subunit | 6.5 ± 1.2 | 2.5E-05 |
| Mbar A 0894 | methyl-coenzyme M reductase gamma subunit | 4.2 ± 0.7 | 3.4E-05 |
| Mbar A 0895 | methyl-coenzyme M reductase subunit C | 7.1 ± 1.1 | 6.7E-05 |
| Mbar A 0896 | methyl-coenzyme M reductase subunit D | 6.4 ± 1.3 | 3.1E-06 |
| Mbar A 0897 | methyl-coenzyme M reductase beta subunit | 6.6 ± 1.1 | 7.9E-05 |
| **Formate to CH_4_ (2 genes)** | |  |  |
| Mbar A 1561 | formate dehydrogenase major subunit | 4.0 ± 1.2 | 5.6E-03 |
| Mbar A 1562 | formate dehydrogenase, beta subunit | 4.6 ± 1.2 | 5.9E-04 |
| A gene with an absolute value of the fold-change ≥ 1.2^†^ and a *p-*value < 0.05^*^ is considered differentially expressed | | | |

***Additional file 9: Gene expression of the selected M. mazei (MM) genes in response to cocultivation with C. cellulovorans (CC) at mid-exponential growth phase***

| ***MM* pathway**  **and gene ID** | **Description** | **Fold-change**^†^ **(*CC-MM*/*MM*)** | ***p*-value**^*^ |
| --- | --- | --- | --- |
| **Acetoclastic pathway (2 genes)** | |  |  |
| MM 0495 | acetate kinase | -12.3 ± 0.9 | 1.6E-06 |
| MM 0496 | phosphate acetyltransferase | -16.8 ± 2.1 | 1.1E-07 |
| **CO_2_ reduction pathway (3 genes)** | |  |  |
| MM 0055 | formylmethanofuran dehydrogenase subunit F | 2.9 ± 0.2 | 1.1E-05 |
| MM 0058 | formylmethanofuran dehydrogenase subunit G | 1.9 ± 0.6 | 4.3E-03 |
| MM 0059 | formylmethanofuran dehydrogenase subunit B | -1.2 ± 0.2 | 1.9E-01 |
| **CH_4_ formation (2 genes)** | |  |  |
| MM 1241 | methyl-coenzyme M reductase gamma subunit | 1.4 ± 0.2 | 4.6E-03 |
| MM 1244 | methyl-coenzyme M reductase beta subunit | 4.0 ± 0.6 | 2.8E-04 |
| A gene with an absolute value of the fold-change ≥ 1.2^†^ and a *p-*value < 0.05^*^ is considered differentially expressed | | | |


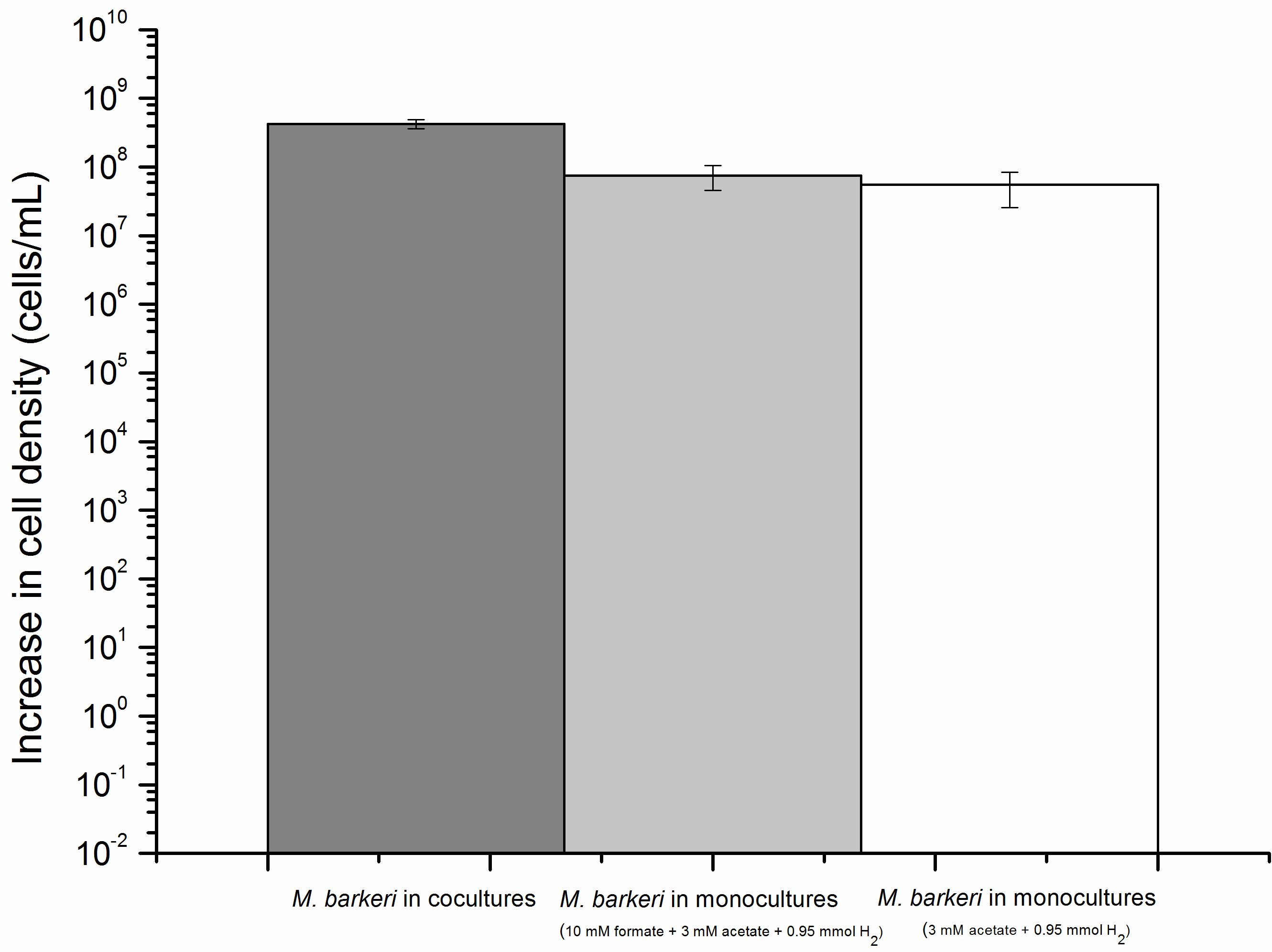


**Additional file 10:** Comparison of the increase in cell density over the course of the experiment. Cell density at time zero was about 3.0 × 10^7^ cells/mL for the experiment
